# Clinical longevity of intracoronal restorations made of gold, lithium disilicate, leucite, and indirect resin composite: a systematic review and meta-analysis

**DOI:** 10.1007/s00784-023-05050-x

**Published:** 2023-08-19

**Authors:** R. A. Bresser, J. W. Hofsteenge, T. H. Wieringa, P. G. Braun, M. S. Cune, M. Özcan, M. M. M. Gresnigt

**Affiliations:** 1 Department of Restorative Dentistry and Biomaterials, Center for Dentistry and Oral Hygiene, University Medical Center Groningen, University of Groningen, Antonius Deusinglaan 1, 9713 AV Groningen, The Netherlands; 2 Department of Epidemiology, University Medical Center Groningen, University of Groningen, Groningen, The Netherlands; 3 Medical Decision Making, Department of Biomedical Data Sciences, Leiden University Medical Center, Leiden, The Netherlands; 4 Central Medical Library, University Medical Center Groningen, University of Groningen, Groningen, The Netherlands; 5 Prosthodontics and Special Dental Care, Department of Oral Maxillofacial Surgery, St. Antonius Hospital, Nieuwegein, The Netherlands; 6 Clinic for Reconstructive Dentistry, Division of Dental Biomaterials, Center of Dental Medicine, University of Zurich, Zurich, Switzerland; 7 Department of Special Dental Care, Martini Hospital, Groningen, The Netherlands

**Keywords:** Adhesive, Inlay, Intracoronal restoration, Partial, Onlay

## Abstract

**Objectives:**

The aim of this systematic review and meta-analysis is to assess the comparative clinical success and survival of intracoronal indirect restorations using gold, lithium disilicate, leucite, and indirect composite materials.

**Material and methods:**

This systematic review and meta-analysis were conducted following the Cochrane Handbook for Systematic Reviews of Interventions and PRISMA guidelines. The protocol for this study was registered in PROSPERO (registration number: CRD42021233185). A comprehensive literature search was conducted across various databases and sources, including PubMed/Medline, Embase, Cochrane Library, Web of Science, ClinicalTrials.gov, and gray literature. A total of 7826 articles were screened on title and abstract. Articles were not excluded based on the vitality of teeth, the language of the study, or the observation period. The risk difference was utilized for the analyses, and a random-effects model was applied. All analyses were conducted with a 95% confidence interval (95% CI). The calculated risk differences were derived from the combined data on restoration survival and failures obtained from each individual article. The presence of heterogeneity was assessed using the *I*^2^ statistic, and if present, the heterogeneity of the data in the articles was evaluated using the non-parametric chi-squared statistic (*p* < 0.05).

**Results:**

A total of 12 eligible studies were selected, which included 946 restorations evaluated over a minimum observation period of 1 year and a maximum observation period of 7 years. Results of the meta-analysis indicated that intracoronal indirect resin composite restorations have an 18% higher rate of failure when compared to intracoronal gold restorations over 5–7 years of clinical service (risk difference =  − 0.18 [95% CI: − 0.27, − 0.09]; *p* = .0002; *I*^2^ = 0%). The meta-analysis examining the disparity in survival rates between intracoronal gold and leucite restorations could not be carried out due to methodological differences in the studies.

**Conclusions:**

According to the currently available evidence, medium-quality data indicates that lithium disilicate and indirect composite materials demonstrate comparable survival rates in short-term follow-up. Furthermore, intracoronal gold restorations showed significantly higher survival rates, making them a preferred option over intracoronal indirect resin-composite restorations. Besides that, the analysis revealed no statistically significant difference in survival rates between leucite and indirect composite restorations. The short observation period, limited number of eligible articles, and low sample size of the included studies were significant limitations.

**Clinical significance:**

Bearing in mind the limitations of the reviewed literature, this systematic review and meta-analysis help clinicians make evidence-based decisions on how to restore biomechanically compromised posterior teeth.

## Introduction

The trend towards prolonging the tooth restoration cycle includes performing minimally invasive restorative procedures to preserve as much enamel as possible. This is achieved by intracoronal indirect restorations, such as inlays and onlays which only replace the lost tooth substrate [1]. Unlike full crown circumferential preparations, intracoronal preparations are less invasive and result in the preservation of a greater amount of healthy tooth substrate [1].

Intracoronal indirect restorations are a viable option for restoring teeth, even in situations with substantial loss of tooth substrate. Indirect restorations have several presumed advantages over direct restorations, including reduced polymerization shrinkage, lower stress within the tooth, and prevention of fracture [2, 3]. These benefits may contribute to better clinical performance and lower annual failure rates compared to direct resin composite restorations [1, 4]. Suitable materials for intracoronal indirect restorations include indirect composite, glass–ceramic, and gold, which may exhibit lower annual failure rates compared to direct restorations made of composite, amalgam, or glass-ionomer materials [4]. However, some studies have found no significant difference in longevity between direct and indirect restorations [5].

Gold alloy restorations have been extensively evaluated in the literature, compared to indirect composite or glass–ceramic restorations. One study found the survival rate of intracoronal gold restorations (G) to be 73.5% (SD 5.4%) after 30 years [6]. Another retrospective clinical study evaluated G with a longer evaluation time and measured a survival rate of 94.1% after over 40 years [7]. Risk factors for failure of G include a lower patient age (HR 0.91; 95%CI 0.85–0.96) and an increased number of restored surfaces (HR 2.55; 95%CI 1.19–5.43). The most commonly reported reasons for G failure are secondary caries and fracture of the tooth [8].

Alternative materials to gold include materials that can be luted adhesively, such as glass–ceramics or indirect composites. In a recent systematic review and meta-analysis, promising results for intracoronal glass–ceramic restorations were reported, with an estimated survival rate of 93% (95% CI [86, 96]) over a 10-year clinical follow-up period, based on 605 restorations [9]. However, some studies have reported lower survival rates for intracoronal glass–ceramic restorations, with a survival rate of 80% after 11 years [10].

Intracoronal indirect composite (IC), lithium disilicate (LD), and leucite (L) restorations are best adhesively luted in an isolated, dry working field due to the hydrophobic nature of resin composites or cements [11, 12]. Isolation may be challenging in cases of deep subgingival contours, where contamination is more difficult to prevent, which can negatively impact the clinical outcome and survival of these restorations [13–16]. Other factors that may influence the performance of these restorations include simplified adhesive systems, non-adhesive luting, patient-, restoration-, and operator-related factors [13, 17]. It should be noted that partial adhesive indirect restorations require a more complex operative procedure and are more time-consuming compared to gold restorations, which are predominantly conventionally cemented [18].

Despite the declining patient acceptance of gold restorations [19], they are still widely used and the subject of ongoing clinical research [20, 21]. However, increasing esthetic demands and changing indications among dentists have led to an increase in the use of tooth-colored materials instead of gold [22]. While there have been clinical studies evaluating the survival rate and clinical success of indirect composite, lithium disilicate, leucite, and gold restorations separately, very few have compared them [19]. As a result, there is a lack of effective measures to quantify the difference in longevity between these materials. The objective of this systematic review and meta-analysis is to examine whether lithium disilicate, leucite, and indirect composite materials can achieve comparable success and survival rates to those of partial gold restorations. It also aims to compare the success and survival rates, as well as the quality, of these materials in the posterior region over time.

## Methods

This systematic review and meta-analysis were conducted following the Cochrane Handbook for Systematic Reviews of Interventions [23] and Preferred Reporting Items for Systematic Review and Meta-Analyses (PRISMA) guidelines [24]. The protocol for this study was registered in PROSPERO (registration number: CRD42021233185).

### Search strategy

The PICOS (Population, Intervention, Comparison, Outcome, Study design) question was initially defined in order to formulate the search strategy, as shown in Table 1. An extensive search was carried out across multiple databases, including those that encompass “grey literature.” The sources used to identify published studies for the systematic review were as follows: PubMed/Medline, Embase (Elsevier platform), Cochrane Library (CENTRAL), Web of Science (Clarivate platform), and the ClinicalTrials.gov study register.

**Table 1:** PICOS question to define the search strategy. *Without a restriction on a minimum observation period

P (population)	Patients in need of an indirect intracoronal restoration
I (intervention)	Gold, resin composite, leucite reinforced or lithium disilicate intracoronal restoration
C (comparison)	Gold, resin composite, leucite reinforced or lithium disilicate intracoronal restoration
O (outcome and study design)	Survival and success rate
S (study type)	Randomized clinical trials (RCTs) and clinical follow-up studies*

The search strategies were developed using a combination of subject headings, free text terms, and the syntax specific to each database. The search included terms related to patients, interventions, and outcomes, as well as criteria for the selection of study types (excluding animal and in vitro studies). No restrictions were placed on the searches, such as date, language, or abstract availability. The translation of the PubMed search strategy to other databases was performed manually, without the use of automated translation software. The search strategy was reviewed by experts in the library literature search. The initial searches were conducted on May 12th, 2021, in all databases and study registers. All searches were repeated and additional articles were screened prior to submission of the manuscript to the journal on January 10th, 2023 [25]. The search strategies for all databases are presented in Table 2.

**Table 2:** Search strategy for each database

Search strategy PubMed	(“Crowns”[Mesh] OR crown*[tiab] OR (“Dental Restoration, Permanent”[Mesh] OR (dental[tiab] OR partial*[tiab] OR coverage*[tiab] OR permanent[tiab] OR composite*[tiab] OR temporar*[tiab] OR provisional*[tiab]) AND (indirect[tiab] OR partial[tiab] OR CEREC[tiab]) AND (restoration*[tiab] OR prothes*[tiab] OR repair*[tiab])) OR inlay*[tiab] OR onlay*[tiab] OR overlay*[tiab] OR ( "Dental Bonding"[Mesh] OR (dental[tiab] OR dentin[tiab]) AND (bonding*[tiab] OR curing*[tiab] OR cure*[tiab] OR “self-curing*”[tiab] OR “self-cure*”[tiab] OR “light-curing*”[tiab] OR “light cure*”[tiab] OR “chemical cure*”[tiab] OR “chemical curing*”[tiab] OR sealing*[tiab])) OR cementation*[tiab] OR (cusp*[tiab] AND coverage*[tiab]) OR IDS[tiab] OR DDS[tiab]) NOT (“Dental Implants”[Mesh] NOT “Dental Restoration, Permanent”[Mesh]) AND (“Gold Alloys”[Mesh] OR “gold alloy*”[tiab] OR (cast*[tiab] AND gold*[tiab]) OR “Dental Porcelain”[Mesh] OR “dental porcelain*”[tiab] OR ceramic*[tiab] OR “IPS Empress”[tiab] OR “lithia disilicate”[Supplementary Concept] OR “lithia disilicate*”[tiab] OR “lithium disilicate*”[tiab] OR “lithium-silicate*”[tiab] OR emax[tiab] OR “IPS-e.max press”[tiab] OR leucite*[tiab] OR LDS[tiab] OR nanoceramic[tiab] OR “Composite Resins”[Mesh] OR “composite resin*”[tiab]) AND (“Dental Restoration Failure”[Mesh] OR “dental restoration failure*”[tiab] OR survival[tiab] OR ((restoration*[tiab] OR clinical*[tiab]) AND (longevity*[tiab])) OR “clinical effectiveness*”[tiab] OR “clinical evaluation*”[tiab] OR “clinical performance*”[tiab] OR “clinical result*”[tiab] OR “clinical outcome*”[tiab] OR “clinical efficac*”[tiab] OR “clinical examination*”[tiab] OR “Treatment Outcome”[Mesh] OR “treatment outcome*”[tiab] OR “treatment effectiveness*”[tiab] OR “treatment efficac*”[tiab] OR “success rate*”[tiab] OR USPHS[tiab]) AND (“Randomized Controlled Trial”[Publication Type] OR “Controlled Clinical Trial”[Publication Type] OR randomized[tiab] OR “Clinical Trials as Topic”[Mesh:NoExp] OR randomly[tiab] OR trial[ti] OR “intervention study”[tiab] OR “Prognosis”[Mesh] OR prognos*[tiab] OR prospectiv*[tiab] OR retrospectiv*[tiab] OR “Follow-Up Studies”[Mesh] OR “follow-up”[tiab] OR followup[tiab] OR “followed-up”[tiab] OR longitudinal*[tiab] OR predict*[tiab] OR associat*[tiab] OR relationship*[tiab] OR “Comparative Study”[Publication Type] OR comparative[tiab] OR “Evaluation Study”[Publication Type] OR evaluation[tiab] OR “Survival Analysis”[Mesh] OR “survival analysis”[tiab]) NOT (“In Vitro Techniques”[Mesh]) NOT (“Animals”[Mesh] NOT “Humans”[Mesh])
Search Strategy Embase	(“tooth crown “/exp OR crown*:ti,ab OR “dental inlay “/exp OR (“dental restoration “/exp OR (“dental”:ti,ab OR “partial*”:ti,ab OR “coverage*”:ti,ab OR “permanent”:ti,ab OR “composite*”:ti,ab OR “temporar*”:ti,ab OR “provisional*”:ti,ab) AND (“indirect”:ti,ab OR “partial”:ti,ab OR “CEREC”:ti,ab) AND (“restoration*”:ti,ab OR “prothes*”:ti,ab OR “repair*”:ti,ab)) OR “inlay*”:ti,ab OR “onlay*”:ti,ab OR “overlay*”:ti,ab OR “occlusal veneer*”:ti,ab OR ( “dental bonding “/exp OR (“dental”:ti,ab OR “dentin”:ti,ab) AND (“bonding*”:ti,ab OR “curing*”:ti,ab OR “cure*”:ti,ab OR “self-curing*”:ti,ab OR “self-cure*”:ti,ab OR “light-curing*”:ti,ab OR “light cure*”:ti,ab OR “chemical cure*”:ti,ab OR “chemical curing*”:ti,ab OR “sealing*”:ti,ab)) OR “cementation*”:ti,ab OR (“cusp*”:ti,ab AND “coverage*”:ti,ab) OR “IDS”:ti,ab OR “DDS”:ti,ab) NOT (“tooth implant”/exp NOT “dental restoration”/exp) AND (“gold alloy”/exp OR “gold alloy*”:ti,ab OR (“cast*”:ti,ab AND “gold*”:ti,ab) OR “dental ceramics”/exp OR “dental porcelain*”:ti,ab OR “ceramic*”:ti,ab OR “IPS Empress”:ti,ab OR “lithia disilicate”/exp OR “lithia disilicate*”:ti,ab OR “lithium disilicate*”:ti,ab OR “lithium-silicate*”:ti,ab OR “emax”:ti,ab OR “IPS-e.max press”:ti,ab OR “leucite*”:ti,ab OR “LDS”:ti,ab OR “nanoceramic”:ti,ab OR “composite resin*”:ti,ab) AND (“dental restoration”/exp OR “dental restoration failure*”:ti,ab OR “survival”:ti,ab OR ((“restoration*”:ti,ab OR “clinical*”:ti,ab) AND (“longevity*”:ti,ab)) OR “clinical effectiveness*”:ti,ab OR “clinical eva sinds te tijd die tussen jullie en de search van vandaag zit. luation*”:ti,ab OR “clinical performance*”:ti,ab OR “clinical result*”:ti,ab OR “clinical outcome*”:ti,ab OR “clinical efficac*”:ti,ab OR “clinical examination*”:ti,ab OR “treatment outcome”/exp OR “treatment outcome*”:ti,ab OR “treatment effectiveness”:ti,ab OR “treatment efficac*”:ti,ab OR “success rate*”:ti,ab OR “USPHS”:ti,ab) AND (“clinical trial”/exp OR “intervention study”:ti,ab OR “randomized”:ti,ab OR “randomly”:ti,ab OR “trial”:ti OR “prognosis”/exp OR “prospectiv*”:ti,ab OR “retrospectiv*”:ti,ab OR “follow up”/exp OR “follow-up”:ti,ab OR “followup”:ti,ab OR “followed-up”:ti,ab OR “longitudinal*”:ti,ab OR “predict*”:ti,ab OR “prognos*”:ti,ab OR “associat*”:ti,ab OR “relationship*”:ti,ab OR “comparative effectiveness”/exp OR “comparative”:ti,ab OR “evaluation study”/exp OR “evaluation”:ti,ab OR “survival analysis”/exp) NOT (“in vitro study”/exp) NOT (“animal”/exp NOT “human”/exp)
Search strategy Web of Science	TS = ((“crown*” OR ((“dental” OR “partial*” OR “coverage*” OR “permanent” OR “composite*” OR “temporar*” OR “provisional*”) AND (“indirect” OR “partial” OR “CEREC”) AND (“restoration*” OR “prothes*” OR “repair*”)) OR “inlay*” OR “onlay”* OR “overlay*” OR “occlusal veneer*” OR ((“dental” OR “dentin”) AND (“bonding*” OR “curing*” OR “cure*” OR “self-curing*” OR “self-cure*” OR “light-curing*” OR “light cure*” OR “chemical cure*” OR “chemical curing*” OR “sealing*”)) OR “cementation*” OR (“cusp*” AND “coverage*”) OR “IDS” OR “DDS”) AND (“gold alloy*” OR (“cast*” AND “gold*”) OR “dental porcelain*” OR “ceramic*” OR “IPS Empress” OR “lithia disilicate*” OR “lithium disilicate*” OR “lithium-silicate*” OR “emax” OR “IPS-e.max press” OR “leucite*” OR “LDS” OR “nanoceramic” OR “composite resin*”) AND (“dental restoration failure*” OR “survival” OR ((“restoration*” OR “clinical*”) AND (“longevity*”)) OR “clinical effectiveness*” OR “clinical evaluation*” OR “clinical performance*” OR “clinical result*” OR “clinical outcome*” OR “clinical efficac*” OR “clinical examination*” OR “treatment outcome*” OR “treatment effectiveness*” OR “treatment efficac*” OR “success rate*” OR “USPHS”) AND (“randomized” OR “randomly” OR “trial” OR “intervention study” OR “prognos*” OR “prospectiv*” OR “retrospectiv*” OR “follow-up” OR “followup” OR “followed-up” OR “longitudinal*” OR “predict*” OR “associat*” OR “relationship*” OR “comparative” OR “evaluation” OR “survival analysis”))
Search strategy Cochrane Database	([mh “Crowns”] OR “crown*”:ti,ab OR ([mh “Dental Restoration, Permanent”] OR (“dental”:ti,ab OR “partial*”:ti,ab OR “coverage*”:ti,ab OR “permanent”:ti,ab OR “composite*”:ti,ab OR “temporar*”:ti,ab OR “provisional*”:ti,ab) AND (“indirect”:ti,ab OR “partial”:ti,ab OR “CEREC”:ti,ab) AND (“restoration*”:ti,ab OR “prothes*”:ti,ab OR “repair*”:ti,ab)) OR “inlay*”:ti,ab OR “onlay*”:ti,ab OR “overlay*”:ti,ab OR “occlusal veneer*”:ti,ab OR ( [mh “Dental Bonding”] OR (“dental”:ti,ab OR “dentin”:ti,ab) AND (“bonding*”:ti,ab OR “curing*”:ti,ab OR “cure*”:ti,ab OR “self-curing*”:ti,ab OR “self-cure*”:ti,ab OR “light-curing*”:ti,ab OR “light cure*”:ti,ab OR “chemical cure*”:ti,ab OR “chemical curing*”:ti,ab OR “sealing*”:ti,ab)) OR “cementation*”:ti,ab OR (“cusp*”:ti,ab AND “coverage*”:ti,ab) OR “IDS”:ti,ab OR “DDS”:ti,ab) NOT ([mh “Dental Implants”] NOT [mh “Dental Restoration, Permanent”]) AND ([mh “Gold Alloys”] OR “gold alloy*”:ti,ab OR (“cast*”:ti,ab AND “gold*”:ti,ab) OR [mh “Dental Porcelain”] OR “dental porcelain*”:ti,ab OR “ceramic*”:ti,ab OR “IPS Empress”:ti,ab OR “lithia disilicate*”:ti,ab OR “lithium disilicate*”:ti,ab OR “lithium-silicate*”:ti,ab OR “emax”:ti,ab OR “IPS-e.max press”:ti,ab OR “leucite*”:ti,ab OR “LDS”:ti,ab OR “nanoceramic”:ti,ab OR [mh “Composite Resins”] OR “composite resin*”:ti,ab) AND ([mh “Randomized Controlled Trial”] OR [mh “Controlled Clinical Trial”] OR “randomized”:ti,ab OR [mh “Clinical Trials as Topic”] OR “randomly”:ti,ab OR “trial”:ti OR “intervention study”:ti,ab OR [mh “Prognosis”] OR “prognos*”:ti,ab OR “prospectiv*”:ti,ab OR “retrospectiv*”:ti,ab OR [mh “Follow-Up Studies”] OR “follow-up”:ti,ab OR “followup”:ti,ab OR followed-up:ti,ab OR “longitudinal*”:ti,ab OR “predict*”:ti,ab OR “associat*”:ti,ab OR “relationship*”:ti,ab OR [mh “Comparative Study”] OR “comparative”:ti,ab OR [mh “Evaluation Study”] OR “evaluation”:ti,ab OR [mh “Survival Analysis”] OR “survival analysis”:ti,ab) NOT ([mh “In Vitro Techniques”]) NOT ([mh “Animals”] NOT [mh “Humans”])
Search terms used in databases with “grey literature”	“dental restoration”

Additionally, various sources of “grey literature” and unpublished studies were searched on April 5th, 2022, using the term “dental restoration” (Table 2). The same eligibility criteria applied to the published literature were used in the search for these studies. However, no eligible publications were found that related to the research question and were therefore excluded from the analysis. The sources used to identify studies for the systematic review included the following: OpenGrey, Cochrane Trial Register (CENTRAL), Clinicaltrials.gov, and NARCIS–database for Dutch theses.

A supplementary search was conducted by manually reviewing the reference lists of all systematic reviews found in the initial search results. No additional articles were discovered that were eligible for inclusion in this systematic review. The identified records were managed using EndNote software, and duplicates were removed using the Bramer method [26].

### Study selection

The titles and abstracts of studies identified in the search were independently screened in duplicate by two authors (JWH and RAB). All potentially relevant studies were then retrieved for full-text screening, which was performed by the same two authors, again in duplicate and independently of each other. The degree of agreement in full-text screening was assessed using Cohen’s Kappa [27]. In cases of disagreement between the authors, the issue was resolved through discussion. If the discussion was not sufficient to resolve the disagreement, a final decision was made by a third author (MMMG). The reference lists of the included studies were also reviewed to ensure that no eligible and relevant studies had been missed.

### Eligibility criteria

Publications were considered eligible for inclusion in the systematic review if they met the following criteria:

- 1. They were randomized controlled trials or retro- and prospective studies that evaluated permanent posterior teeth that required or possessed an intracoronal restoration and assessed the survival of gold, lithium disilicate, leucite, and/or resin composite indirect intracoronal restorations in the posterior region.
- 2. The success of a restoration was defined as the absence of clinical intervention.
- 3. The restoration was defined as a success failure in case of chipping, hypersensitivity, endodontic treatment, or small repair.
- 4. A total failure of a restoration was defined as a fracture of the restoration or tooth or secondary caries.
- 5. Failures due to extraction due to severe periodontal breakdown were censored.

### Exclusion criteria

Publications were excluded from the review if they met any of the following criteria:

1. Evaluated a single material (gold, lithium disilicate, leucite, or indirect composite) exclusively
2. Systematic reviews, case reports, conference abstracts, viewpoints or opinion papers, or protocols. Systematic reviews were separately reviewed for potential missed eligible publications
3. Only described conventional circumferential restorations, fixed dental prostheses, endocrowns, or implant restorations
4. Only included subjects with intracoronal restorations in deciduous teeth and/or in the anterior region of the oral cavity
5. Polymeric infiltrated ceramic network (PICN) material was excluded from analysis.

The review did not consider the vitality of posterior teeth or the language or follow-up time of the studies as inclusion or exclusion criteria.

### Data extraction and collection

The data extraction of the included full-text publications was carried out independently and in duplicate by two authors (JWH and RAB). An Excel sheet was created for data extraction, study quality assessment, and evidence synthesis. The sheet included the following information:

- General trial information: author, year, title, journal, country of study, language, patient population inclusion and exclusion criteria, type of study, setting, number of patients and restorations, gender, age, follow-up time, premolars or molars, inlays or onlays, one or multiple surfaces, mandible or maxilla, dropouts
- Intervention characteristics: type of restorative material, immediate dentin sealing (IDS), pretreatment of the restoration and tooth, cementation material, isolation, liner
- Outcome data: survival (n), Kaplan Meier probability, failures, time of failure, and type of failure

Additionally, the quality of the surviving restorations was assessed as a secondary outcome (clinical performance).

The method of qualitative assessment was noted along with its corresponding outcome data (modified United States Public Health Service (USPHS) criteria, Federation Dentaire Internationale (FDI) criteria, California Dental Association (CDA) criteria). In order to make the variables comparable and useful for meta-analysis (dichotomous), all acceptable outcomes were clustered. The modified USPHS “alpha” and “bravo” scores were considered equivalent to a score of “1,” “2,” and “3” on the FDI criteria and “excellent” and “sierra” on the CDA criteria, as demonstrated in Table 3 [28, 29].

**Table 3:** Dichotomy of results according to the evaluation criteria of the studies

Parameters	USPHS modified criteria		FDI World Federation criteria		CDA criteria	
	Acceptable	Unacceptable	Acceptable	Unacceptable	Acceptable	Unacceptable
Anatomic form	Alpha, Bravo	Charlie, Delta	1, 2, 3	4,5	Excellent, Sierra	Tango, Victor
Color match	Alpha, Bravo	Charlie, Delta	1, 2, 3	4,5	Excellent, Sierra	Tango, Victor
Surface texture	Alpha, Bravo	Charlie, Delta	1, 2, 3	4,5	Excellent, Sierra	Tango, Victor
Marginal adaptation	Alpha, Bravo	Charlie, Delta	1, 2, 3	4,5	Excellent, Sierra	Tango, Victor
Marginal discoloration	Alpha, Bravo	Charlie, Delta	1, 2, 3	4,5	Excellent, Sierra	Tango, Victor

If data were missing in the full-text publication, the corresponding author was contacted to provide clarification or additional data. If the author did not respond after two reminders, the study was excluded from the quantitative analysis.

In the event of disagreement in the data collected by the two authors, the issue was resolved through discussion. If the discussion did not resolve the disagreement, a final decision was made by a third researcher (MMMG). The final data extraction sheet was used to import the data into Revman 5.4 software (Review Manager v. 5, The Cochrane Collaboration; Copenhagen, Denmark) for meta-analysis.

### Assessment of risk of bias in included studies

The risk of bias was assessed independently and in duplicate by two authors (JWH and RAB). The risk of bias for retrospective and prospective studies of interventions was evaluated using the Risk of Bias In Non-randomized Studies—of Interventions (ROBINS-I) tool [30], while the risk of bias for randomized clinical trials (RCTs) was assessed using the Cochrane tool for risk of bias in randomized trials (RoB 2 tool) [31]. In case of disagreement in the assessment, the matter was resolved through discussion. If the discussion did not result in consensus, a final decision was made by a third author (MMMG). The overall risk of bias judgement was determined by the highest risk among all domains. The Grading of Recommendations Assessment, Development, and Evaluation (GRADE) system was used to evaluate the evidence produced by this review [32]. The body of evidence was rated independently and in duplicate by two reviewers (JWH and RAB).

### Investigation of heterogeneity, data synthesis and subgroup analysis

Meta-analyses were conducted after evaluating clinical, methodological, and statistical heterogeneity. The presence of heterogeneity was assessed using the *I*^2^ statistic, and if present, the heterogeneity of the data in the articles was evaluated using the non-parametric chi-squared statistic (*p* < 0.05). Due to the methodological heterogeneity, randomized clinical trials and retrospective and prospective studies were presented in separate forest plots.

The meta-analyses aimed to determine the significant difference in survival and success outcomes between gold, lithium disilicate, leucite, and indirect composite. The analyses were based on risk differences as some studies did not have any events, and it was not possible to detect an effect with odds ratios or risk ratios in studies without events. To maintain consistency, all analyses were performed using the risk difference. A random-effects model was applied, and all analyses were conducted with a 95% confidence interval (95% CI). In cases of heterogeneity, subgroup analyses were performed, when feasible, to identify the source and location of the heterogeneity.

## Results

### Study selection

**Fig. 1:**
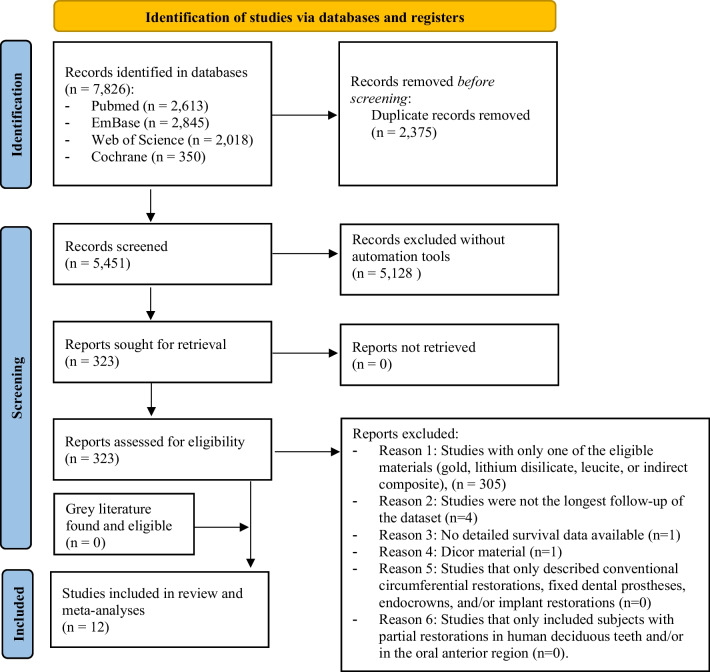
Flowchart of the study selection based on the Preferred Reporting Items for Systematic Reviews and Meta-Analyses of the systematic review

The initial search resulted in 7826 articles, of which duplicates were removed. The remaining articles underwent title and abstract screening, followed by full-text analysis to assess their eligibility. The inter-rater reliability of the full-text screening between two independent researchers was rated as excellent, with a kappa statistic of 0.87 [24]. One eligible article could not be included due to the lack of detailed survival data [20]. Finally, 12 articles were included in the systematic review and meta-analyses (Fig. 1). The 12 studies included in the meta-analyses comprised 5 RCTs, 3 prospective clinical trials, and 4 retrospective observational studies.

### Risk of bias within studies

**Fig. 2:**
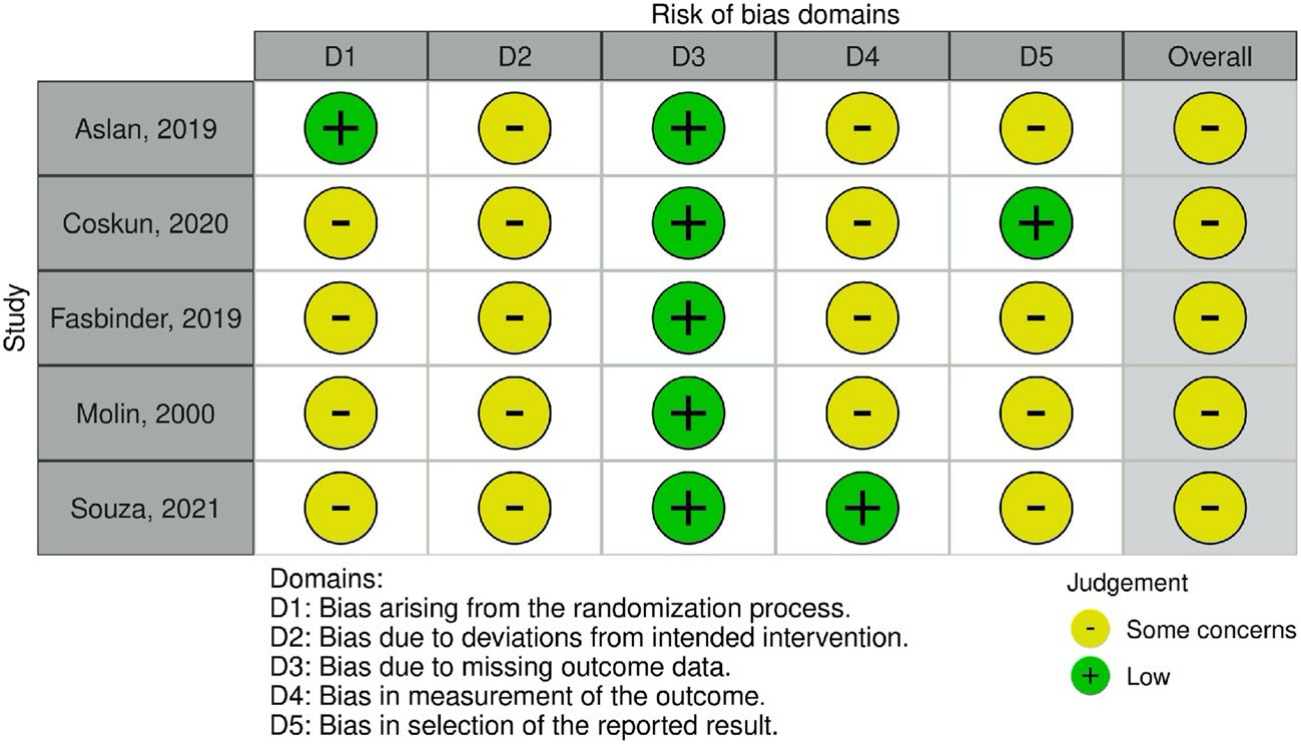
Risk of bias assessment in RCT studies using the Cochrane RoB2 tool

**Fig. 3:**
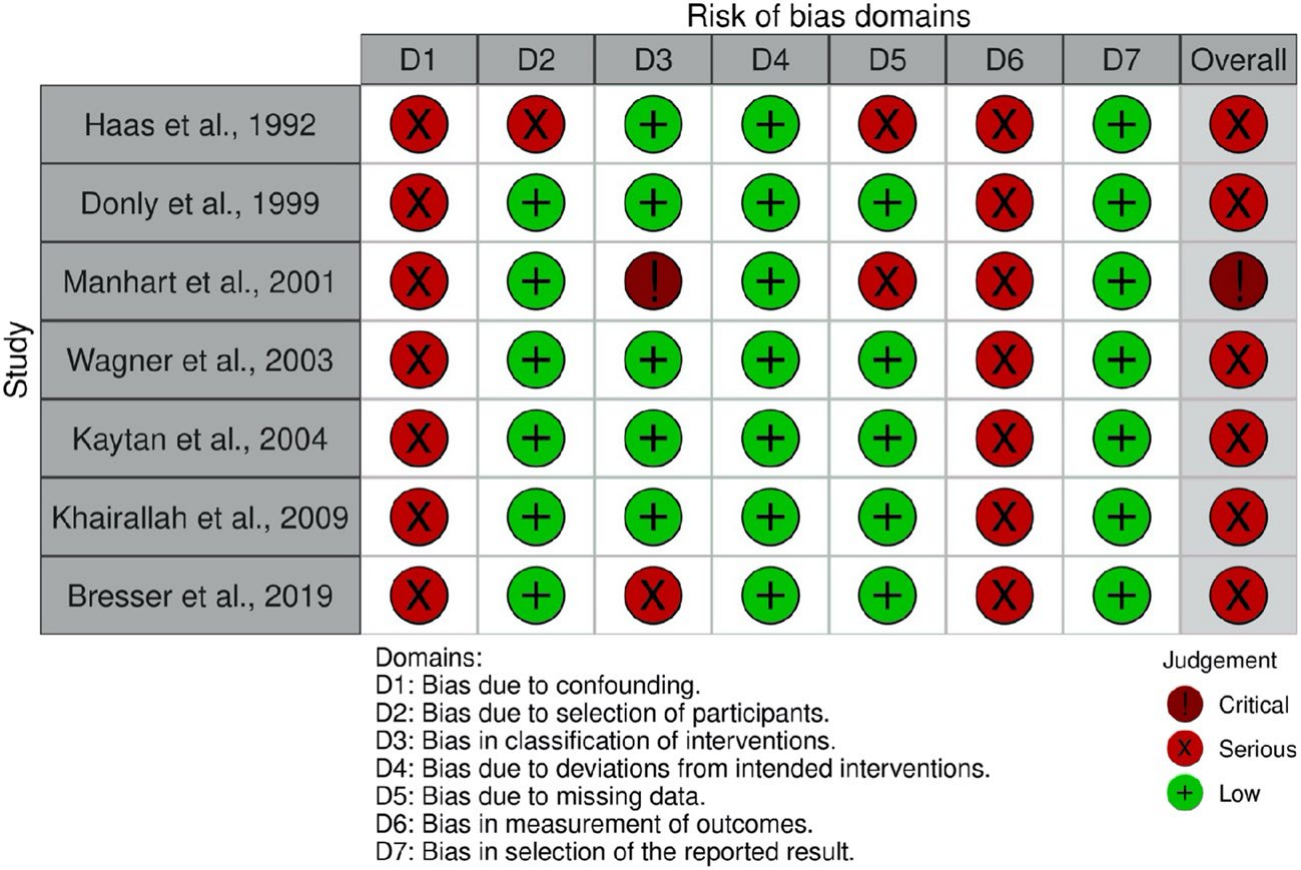
Risk of bias assessment in observational studies using the Cochrane ROBINS-I tool

The risk of bias was evaluated in all 12 relevant studies. The assessment revealed that all RCTs presented some concerns regarding the risk of bias, but none of them was at a serious risk of bias (Fig. 2). In contrast, all retrospective and prospective studies were considered to be at a serious risk of bias, with one study having a critical risk of bias (Fig. 3) [33]. The study by Manhart et al. (2001) was deemed to have induced bias in the classification of the intervention domain by assigning larger restorations to the L group and smaller restorations to the IC group.

### Study characteristics

The characteristics of the included studies are summarized in Table 4. In total, 946 restorations were evaluated over a maximum evaluation period of 7 years, including 140 G, 219 LD, 204 L, and 383 IC restorations. The selected studies were published between 1991 and 2021. One study [20], which evaluated the survival rate of various ceramic and gold restorations meeting various inclusion criteria, was excluded from the meta-analyses as it did not present the number of failures for lithium disilicate and leucite restorations, and further inquiries with the authors did not yield any additional information. The types of failures are specified in the table of characteristics, with the most frequent types of failures being fracture of the restoration, secondary caries, and endodontic treatment.

**Table 4:** Overview of the characteristics of the included studies

Study	Setting	Materials	Evaluation criteria	No of patients	No of teeth	Type of restoration	Evaluated teeth. Restoration material	Cementation material	Follow-up (y)	Dropout (%)	Survival (%)	Type of failure
RCT												
Aslan et al. (2019)	University	LD. IC	Modified USPHS	35 M: 12 W: 23	75 Pr: 15 Mo: 60	Inlay: 22 Onlay: 53	25 LD: IPS e.max CAD	DC. Variolink Esthetic	1^ mn^	0	100	
							25 GC: lithium aluminosilicate	DC. Variolink Esthetic	1^ mn^	0	100	
							25 IC: Cerasmart	DC. Variolink Esthetic	1^ mn^	0	100	
Coşkun et al. (2020)	University	LD. IC	Modified USPHS	14 M: 8 F: 6	60 Pr: 10 Mo: 60	Inlay: 4 Onlay: 56	30 LD: IPS e.max CAD	DC. Variolink Esthetic	2^ mn^	0	100	
							30 IC: Cerasmart	DC. Variolink Esthetic	2^ mn^	0	100	
Souza et al. (2021)	University	LD. IC	FDI	20 M: 15 F: 5	Pr: 4 Mo: 16	Onlay: 40	20 LD: IPS e.max CAD	DC. RelyX Ultimate	1^ mn^	0	100	
					Pr: 0 Mo:20		20 IC: Lava Ultimate CAD	DC. RelyX Ultimate	1^ mn^	0	100	
Fasbinder et al. (2020)	University	L. IC	Modified USPHS	86 M: 30 F: 56	120 Pr: 38 Mo: 82	Onlay: 120	60 L: IPS Empress CAD	DC. RelyX ultimate. Variolink II	5^ mn^	0	91.7	4 restoration fracture 1 restoration chipping
							60 IC: Lava Ultimate CAD	DC. RelyX ultimate. Variolink II	5^ mn^	0	90	2 endodontic treatment 2 tooth fracture 1 restoration fracture 1 restoration chipping
Molin & Karlsson (2000)	University	L. G	CDA	20 M: 9 F: 11	20 Pr: 11 Mo: 9	Inlay: 20 Surfaces: 2: 9 3: 11	20 L: IPS Empress	DC	5^ mn^	0	80	4 restoration fracture
					20 Pr: 14 Mo: 6	Inlay: 20 Surfaces: 2: 4 3: 16	20 G	Zinc Phosphate	5^ mn^	0	95	1 hypersensitivity
Prospective												
Donly et al. (1999)	University	G. IC	USPHS	18	Pr: 5 Mo: 13	Inlay: 11 Onlay: 7	18 G	Zinc Phosphate	7^ mn^	0	83.3	x secondary caries x tooth fracture x restoration fracture
					Pr: 18 Mo: 18	Inlay: 32 Onlay: 4	36 IC	DC	7^ mn^	0	75	x secondary caries x fracture of tooth-restoration interface
Manhart et al. (2001)	University. Students	L. IC	Modified USPHS	45	21 Pr: 8 Mo: 13	Inlay: 21. Surfaces: 1: 1 2: 4 > 2: 16	21 L: IPS Empress	DC. Sono Cem & Variolink Ultra	3^ mn^	0	100	
					37 Pr: 23 Mo: 13	Inlay: 37. Surfaces: 1: 4 2: 19 > 2: 14	37 IC: Tetric. Blend-a-Lux & Pertac-Hybrid unifill	DC. Sono Cem & Variolink Ultra	3^ mn^	0	89.2	
Kaytan et al. (2005)	University	L. IC	Modified USPHS	47 M: 14 F: 33	Mo: 87	Inlay: 87	43 L: IPS Empress	DC. Variolink II Low	2^ mn^	7.4	97.7	1 endodontic treatment
							44 IC: Solidex	DC. Variolink II Low	2^ mn^		100	
Khairallah et al. (2009)	University	L. IC	Modified USPHS	15	18 Pr: 6 M: 12	Inlay: 18. Surfaces: 1: 2 2: 3 > 2: 13	18 L: IPS Empress	DC. RelyX ARC	6.25^ mn^	0	94.40	2 marginal opening 1 endodontic treatment 1 restoration fracture
					18 Pr: 6 M: 12	Inlay: 18. Surfaces: 1: 3 2: 12 > 2: 21	18 IC: Targis	DC. RelyX ARC	6.25^ mn^	0	100	
Retrospective												
Haas et al. (1992)	University	IC. G	None	73	NS	NS	30 G: Degulor C	Microfill Pontic C	5	NS	100	NS
							30 G: Degulor C	Zinc Phosphate. Mizzy-Fleck’s	5		100	
							30 IC: Coltene	Duo Cement	5		80	
							30 IC: Kulzer	Microfill Pontic C	2		80	
							30 GC: Dicor	Tulux Cement	5		93.33	
Wagner and Schmalz (2003)	University	L. G	Modified USPHS	64	84 Pr: 16 Mo: 68	Onlay: 84	42 L: IPS Empress	DC	5.25^md^ (1–6)	0	96	2 restoration fracture
							42 Gold	Phosphate cement	4.75^md^ (0.25–13.08)	0	96	2 extraction for periodontal reasons
Bresser et al. (2019)	Private practice	LD. IC	Modified USPHS	120 M: 42 F:78	197 Pr: 54 Mo: 143	Inlays: 9 Onlays: 188	144 LD: IPS e.max Press	Heated composite. Estelite Σquick	4.8^ mn^	0	96.5	2 secondary caries 1 restoration fracture
							53 IC: Adoro	Heated composite. Estelite Σquick	4.8^ mn^	0	94.3	3 secondary caries 1 endodontic treatment 1 severe periodontal breakdown

*NS*, non-specified; *LD*, lithium disilicate; *L*, leucite; *GC*, glass ceramic; *IC*, indirect resin composite; *G*, gold; *DC*, dual cure; M*,* male; *F* = female; *Pr*, premolar; *Mo*, molar; ^*md*^, median; ^*mn*^, mean

### Meta-analysis of difference in survival and success failures

#### Survival and success of intracoronal gold versus indirect resin composite restorations

**Fig. 4:**
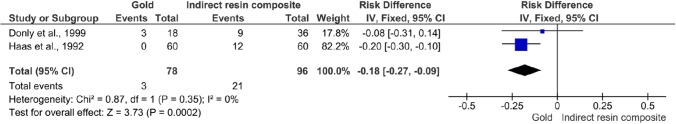
Forest plot of the survival of gold versus indirect resin composite intracoronal restorations in retro- and prospective studies

The meta-analysis conducted on intracoronal gold and indirect resin composite restorations revealed a statistically significant difference in survival rates (risk difference =  − 0.18 [95% CI: − 0.27, − 0.09]; *p* = 0.0002; *I*^2^ = 0%, Fig. 4). The findings suggest that over a clinical service period of 5–7 years, intracoronal indirect resin composite restorations have an 18% higher likelihood of failure compared to intracoronal gold restorations. Notably, all failures were considered survival failures, and no distinction was made between survival and success failures.

#### Survival of intracoronal lithium disilicate and indirect resin composite restorations

**Fig. 5:**
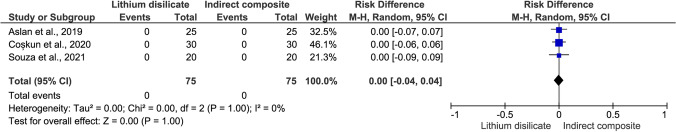
Forest plot of the survival of lithium disilicate versus indirect resin composite intracoronal restorations in RCT studies

The analysis revealed no statistically significant difference in survival rates between LD and IC treatments (Risk difference =  − 0.00 [95% confidence interval: − 0.04, 0.04]; *p* = 1.00; *I*^2^ = 0%, Fig. 5). There was no significant risk difference observed among the studies. However, one observational study that compared LD and IC was excluded from the meta-analysis due to methodological heterogeneity [39]. This study reported on 197 restorations over a 5-year clinical service period, with three failures occurring in the LD group (*n* = 144) and five failures occurring in the IC group (*n* = 53). One of the failures in the LD group concerned a fracture of the restoration.

#### Survival of intracoronal leucite and indirect resin composite restorations

**Fig. 6:**
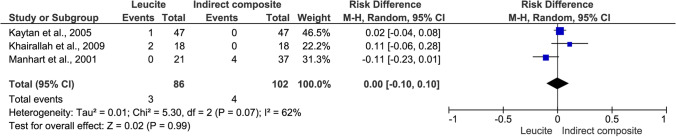
Forest plot of the survival of leucite versus resin composite intracoronal indirect restorations in retro- and prospective studies

The analysis revealed no statistically significant difference in survival rates between L and IC treatments (risk difference = 0.00 [95% confidence interval: − 0.10, 0.10]; *p* = 0.84; *I*^2^ = 48%, Fig. 6). The studies showed moderate heterogeneity, with an *I*^2^ of 62%. A chi-squared test indicated homogeneity between the studies (*X*^2^ (df = 2) = 5.30, *p* = 0.07). Although Manhart et al. (2001) suggested that larger cavities in the L group may have increased the bias risk (Fig. 3), a higher number of failures were observed in the IC group [33]. Three L and two IC restorations were considered success failures (risk difference =  − 0.01 [95% CI: − 0.07, 0.06]; *p* = 0.44; *I*^2^ = 39%). However, one RCT comparing L and IC restorations was excluded from the meta-analysis due to methodological heterogeneity [34]. This study evaluated 120 restorations over a 5-year clinical service period, with five failures observed in the leucite group (*n* = 60) and six failures in the IC group (*n* = 60).

#### Survival and success of intracoronal gold versus leucite restorations

The meta-analysis examining the disparity in survival rates between intracoronal gold and leucite restorations could not be carried out due to methodological differences in the two studies [19, 35]. One RCT study evaluated 40 restorations over a 5-year clinical service period, with four failures observed in the leucite group (*n* = 20) and one failure noted in the gold group (*n* = 20) [35]. All four failures in the leucite group were linked to fractures, while the failure in the gold group was attributed to hypersensitivity. The second study was an observational study that assessed 84 restorations over a period of approximately 5 years of clinical service, with two failures observed in the leucite group (*n* = 42) and two failures recorded in the gold group (*n* = 42) [19]. The two failures in the leucite group were also attributed to fractures, while the two failures in the gold group were censored since they were linked to severe periodontal breakdown.

#### Descriptive analysis of the survival of lithium disilicate versus leucite restorations

Although statistical analysis for these materials was not feasible, a notable difference was observed between all of the LD (*n* = 219) and L (*n* = 204) restorations. The L group had a higher number of failures, predominantly due to fractures, with 11 out of 16 failures being attributed to this cause. In contrast, the LD group had only 3 failures, with only 1 being due to a fracture.

#### Qualitative evaluation assessment for clinical performance

Due to the limited data available, only a meta-analysis to measure the difference in clinical performance between LD and IC restorations could be conducted. However, all restorations were rated as acceptable according to the allocation of Table 2, rendering the purpose of conducting a meta-analysis redundant. Furthermore, insufficient information was available to perform a statistical analysis for other comparisons. Several studies only presented restorations with excellent or equivalent alpha scores, limiting the possible comparisons to only LD and IC restorations [34–36]. Some studies did not provide any qualitative scores [37, 38].

### Quality of evidence

The results of the GRADE evidence profile, as displayed in Tables 5, 6, and 7, indicate that the majority of the evidence in the retro- and prospective studies was of low quality. This was due to the limitations in the study methodology, which resulted in a serious or very serious risk of bias. However, there was no significant evidence of inconsistency, indirectness, or imprecision in the studies.

**Table 5:** GRADE evidence profile table for restoration survival: leucite versus indirect resin composite

Quality assessment							No of restorations		Effect	Quality
No of studies	Design	Risk of bias	Inconsistency	Indirectness	Imprecision	Other considerations	Leucite	Indirect resin composite	Absolute	
3	Prospective studies^1^	Very serious^2^	No serious inconsistency	No serious indirectness	No serious imprecision	None	3/86 (3.5%)	4/102 (3.9%)	4 fewer per 100 (from 4 to 4 fewer)^3^	OOO Very low

Range of follow-up: 4–75 months

^1^Starting from a low-quality level of evidence

^2^Serious or critical risk of bias in Robins-I

^3^Non-significant effect

**Table 6:** GRADE evidence profile table for restoration survival: lithium disilicate versus indirect resin composite

Quality assessment							No of restorations		Effect	Quality
No of studies	Design	Risk of bias	Inconsistency	Indirectness	Imprecision	Other considerations	Lithium disilicate	Indirect resin composite	Absolute	
3	RCT’s^1^	Serious^2^	No serious inconsistency	No serious indirectness	No serious imprecision	None	0/75 (0%)	0/75 (0%)	-	O Moderate

Range of follow-up: 12–24 months

^1^All studies randomized clinical trials, starting from high-quality level of evidence

^2^Some concerns of risk of bias in ROB-2

^3^Non-significant effect

**Table 7:** GRADE evidence profile table for restoration survival: gold versus indirect resin composite

Quality assessment							No of restorations		Effect	Quality
No of studies	Design	Risk of bias	Inconsistency	Indirectness	Imprecision	Other considerations	Gold	Indirect resin composite	Absolute (95% Cl)	
2	Retro- and prospective studies	Serious^2^	No serious inconsistency	No serious indirectness	No serious imprecision	None	3/78 (3.8%)	21/96 (21.9%)	18 fewer per 100 (from 9 to 27 fewer)^3^	OOO Very low

Range of follow-up: 24–84 months

^1^Starting from the low-quality level of evidence

^2^Robins-I shows a serious risk of bias

^3^Significant effect (*p* = 0.00002)

## Discussion

This systematic review and meta-analysis represent the initial comparison of the clinical survival of intracoronal indirect restorations made of gold, lithium disilicate, leucite, and resin composite. This study provides some novel insights into intracoronal indirect adhesive restorations. Within the limitations of this systematic review and meta-analysis, we have found low-quality evidence that favors partial gold over indirect composite restorations. Thus, based on the available data, restorations of gold alloy material can be considered superior over indirect composites on medium- to long-term follow-up. It must however be considered that both articles included in the meta-analysis are at least 20 years old. In the past few decades, many advances were made in the field of adhesive dentistry, and indirect composite materials have been developed over time as well. The application of silica coating, IDS technique, and silane has significantly improved the adhesive protocol for luting indirect restorations to dentin and for adhesive bonding to the resin composite itself [39–45]. The IDS technique increases the adhesion of restorations to dentin but was only used in the adhesive luting process of one of the studies included in the current systematic review and meta-analysis [46]. These adhesive improvements might contribute to better clinical results of indirect composite restorations, and therefore, the provided conclusion should be interpreted with caution.

Furthermore, it was noted that lithium disilicate restorations did not demonstrate significantly better survival rates in contrast to indirect composites, as no cases of failure were observed in any of the randomized controlled trials [47–49] included in the study. Therefore, based on the current data, these materials can be deemed equivalent during short-term follow-up. However, it should be acknowledged that the absence of restoration failure within this limited follow-up duration is not surprising. An additional observational study was included, which could not be incorporated into the meta-analysis due to methodological heterogeneity [46]. This study investigated the survival rates of lithium disilicate and indirect composite restorations over a period of 5 years and found no significant difference between the two materials [46]. However, further research, conducted over a more extended duration, is required to determine which material is superior.

On short- to medium-term follow-up, neither leucite restorations nor indirect composite restorations demonstrated significant superiority. The failures observed across the studies could not be predominantly attributed to a specific restorative material, as they were distributed between the leucite and indirect composite groups.

A meta-analysis could not be performed for the intracoronal gold and leucite restorations due to insufficient data and information, thereby preventing the drawing of any firm conclusions about these materials. The comparison between lithium disilicate and leucite restorations could also not be subjected to statistical analysis, but an interesting observation was made. It was noted that a significant number of leucite failures documented in the reviewed studies were attributed to fractures of the restoration material [19, 34, 35, 38], which has lower flexural strength compared to lithium disilicate [55]. In contrast, the lithium disilicate restorations, for example in the study conducted by Bresser et al. (2019) [46], reported only one fracture. Consequently, the utilization of lithium disilicate is expected to reduce the occurrence of fractures and could extend the survival rates of glass–ceramic restorations.

Gold restorations have a well-established reputation in the literature for their survival rates and clinical performance [7]. The use of resin composite and ceramic materials has increased since their introduction [22]. Adhesive protocols have been developed and improved over time to enhance the adhesive bond strength to tooth enamel and dentin. All four materials, gold, lithium disilicate, leucite, and resin composite, are suitable as intracoronal restorative materials and can be designed to fit the shape of the cavity. This is a distinct advantage over circumferential preparations, as in many cases, only caries lesions and old restorations need to be removed. Conventional cemented gold restorations may require a more aggressive preparation design, sometimes including retentive grooves. Minimizing loss of dental tissue may contribute to a prolonged restoration cycle for teeth [1, 2].

The articles included in the meta-analysis have a wide range of publication dates, which makes comparison and conclusions about these materials more challenging. This is mainly because there are only a few studies that compare two or more materials within the same article, and advancements in restorative dentistry have taken place over the years. Although the studies on gold restorations are at least 20 years old, gold is still used today to restore extensive dental cavities in general dental practice [20, 21]. Therefore, it remains a relevant restorative material. Gold restorations can be placed in less stringent dry conditions, making it a valid alternative to ceramics or indirect composites in cases where rubber dam placement is difficult due to extensive apical restorative outlines.

In several clinical studies, the intaglio surface of leucite restorations was not etched prior to placement [19, 35]. However, in vitro studies have demonstrated that proper etching leads to increased bonding strength [40]. In addition to the IDS technique, there are several other strategies aimed at reducing the rate of adhesive failure, including silica-coating, silane application, ceramic etching, and isolation [14, 50, 51]. A recent systematic review tried to investigate whether the use of rubber dam might be of significant influence on restoration survival [14]. The authors reported that incorporating rubber dam during direct restorative dental treatments may lead to reduced rates of restoration failure within the initial 6-month period after treatment. However, it must be noted that the evidence supporting this finding is of low certainty. The use of rubber dam is believed to prevent contamination by oral fluids and might enhance tensile bond strengths [11, 16, 50, 51]. Despite this potential benefit, the included studies did not always utilize rubber dam, which may have contaminated the dentin and enamel surfaces [19, 34–37, 52].

In addition to differences in adhesive procedures, the reviewed studies also exhibit clinical diversity in other aspects. The size of the restorations, the presence or absence of cusp capping (inlays vs onlays), and other factors contribute to this diversity. Furthermore, all indirect composites were categorized together in the systematic review, although there is a wide range of indirect composite materials with varying characteristics, and no correction was made for this potential source of diversity [56]. Besides that, preparation guidelines for ceramic restorations could also have changed over the years and could have led to different preparation designs [53].

Most of the studies included in this systematic review utilized an etch-and-rinse adhesive system, which typically involved etching followed by a 1-step bonding process [33, 36, 38, 47–49, 52]. One study utilized an IDS layer, which was appropriately treated with silica coating, silane, and adhesive [46]. The survival of adhesively placed restorations is closely linked to the bond strength of the various adhesives employed and could influence the outcome of the meta-analysis.

It is important to note that the majority of studies in this systematic review treated vital teeth, which could influence the outcome of the meta-analysis. Only two out of twelve studies included in this systematic review and meta-analysis addressed the treatment of vital and non-vital teeth. Wagner (2003) treated three non-vital teeth within the 40 gold restorations, but no statements were made regarding their effect on survival. Bresser et al. (2019) included 45 non-vital teeth in the 197 restorations, and their study found no statistically significant influence on survival (*p* > 0.05) [19, 46]. A recent systematic review and meta-analysis on bonded partial indirect posterior restorations showed better survival rates for vital teeth compared to non-vital teeth [54].

Moreover, the results from the current meta-analyses are based on studies that have some potential for bias due to the lack of blinding of personnel, patients, and external examiners. This is partly due to the inherent characteristics of the materials, which make blinding of patients, operators, or outcome assessors impossible. The difference between gold and glass ceramic, in terms of color, is readily apparent, and operators familiar with glass ceramics and resin composite can often distinguish between the materials by visual examination. As a result, it is difficult to conduct a double-blind study.

Additionally, this systematic review is limited by the absence of information regarding the impact of patient-related factors such as socioeconomic status (SES), oral hygiene, caries risk, and occlusal stability on the success and survival of the restorations. Some of the studies included in this systematic review focused primarily on the impact of the material on restoration survival, excluding factors such as bruxism and poor oral hygiene [36, 38, 47, 48]. The influence of caries risk was not evaluated in the studies included, although Molin and Karlsson (2000) noted the plaque index and found no difference in the development of secondary caries between leucite and gold restorations at equivalent levels of plaque index. Studies that did not exclude bruxism and other parafunctions also failed to statistically analyze the impact of bruxism on restoration survival. Interestingly, Molin and Karlsson (2000) observed four restorative fractures with wear facets in the leucite group, indicating that whether a patient has bruxism could indeed be a factor affecting restoration survival [19, 35, 46].

In order to make meaningful conclusions about the behavior of materials over an extended period of time, a more extensive follow-up period is necessary. Despite the fact that most material failures occur after a number of years, long-term follow-up studies are frequently lacking in systematic reviews and meta-analyses. The short follow-up period in many of the studies included in these analyses results in an absence of failure data, making it challenging to formulate statements about long-term outcomes [47–49]. Hence, the validity of the conclusions drawn with regard to these materials over a prolonged evaluation period is questionable. Although some encouraging outcomes are starting to emerge, there are still scarce long-term studies available for adhesive materials such as G and IC, with much still unknown about their behavior after 20 years [9, 55]. This may also account for the lack of differences in survival quality between the RCT studies on lithium disilicate and indirect resin composite restorations, as the meta-analysis only included a maximum follow-up period of 2 years. Ultimately, care should be exercised in the interpretation of the results, due to the risk of bias in the studies included in the meta-analyses and the general low quality of the evidence presented.

Concluding, it is important to note that only a limited number of articles compared the survival rates of two materials and met the criteria for this research question. Thus, future research should aim to evaluate two or more materials simultaneously to facilitate meaningful comparisons. Furthermore, additional long-term studies, studies on newer materials such as lithium disilicate or polymer-infiltrated ceramic network, and studies examining the impact of IDS on survival, may impact the findings of this systematic review [56].

## Conclusion

The results of the current systematic review and meta-analysis demonstrated that neither intracoronal lithium disilicate restorations nor intracoronal indirect composite restorations were inferior to the other. The data suggests that there is medium-quality evidence to support the equivalence of these materials in terms of survival on a short observation period. Furthermore, the analysis revealed low-quality evidence for no statistically significant difference in survival rates between leucite and indirect composite restorations. On the other hand, intracoronal gold restorations showed significantly higher survival rates, making them a preferred option over intracoronal indirect resin-composite restorations. The short follow-up time, limited number of eligible articles, and low sample size of the included studies were significant limitations of the reviewed literature.

## Data Availability

The data that support the findings of this study are available from the corresponding authors upon reasonable request.
